# Clinical Outcomes in Trans-sphenoidal Surgery for Pituitary Neuroendocrine Tumour in Sarawak General Hospital, Malaysia: Microscopic Versus Endoscopic Approach

**DOI:** 10.7759/cureus.111962

**Published:** 2026-07-02

**Authors:** Pei Meng Ng, Albert SH Wong, Ing Ping Tang, Jafri M Abdullah

**Affiliations:** 1 Neurosurgery, Sarawak General Hospital, Kuching, MYS; 2 Otorhinolaryngology, Head and Neck Department, Sarawak General Hospital, Kuching, MYS; 3 Neurosurgery, Universiti Sains Malaysia Health Campus, Kota Bharu, MYS

**Keywords:** adenoma, endoscopic, malaysia, microscopic, outcome, pituitary neuroendocrine tumour, sarawak, transsphenoidal

## Abstract

Purpose: Trans-sphenoidal surgery is the standard approach to provide direct access to pituitary neuroendocrine tumours (PitNET). Reported outcomes for trans-sphenoidal surgical resection of PitNET were heterogeneous. This work aimed to develop local data for the outcomes of trans-sphenoidal surgery, either via a microscopic or endoscopic approach.

Methods: We performed a retrospective review of patients with PitNET treated by trans-sphenoidal surgery at Sarawak General Hospital from 2014-2021. We included only cases treated by the senior author (AWSH) to avoid inter-surgeon variability. Demographic, clinical, radiologic, operative, and postoperative outcome data were analysed.

Results: There were 60 patients, with 35 patients in the microscopic group and 25 in the endoscopic group. The baseline characteristics were comparable. The rate of gross total resection was similar (34.4% vs 44.0%, p=0.459) in both approaches. The endoscopic approach was observed to have a higher proportion of patients with arachnoid breach than the microscopic approach (p=0.010). However, the endoscopic approach had a significantly higher proportion of patients with improved anterior pituitary gland function than the microscopic approach (p=0.006). There was low morbidity, with a meningitis risk of 2.9% in the microscopic approach vs. 4.0% in the endoscopic approach (p>0.950). There was no mortality observed in our study. During follow-up, we found that 83% of patients with residual volume remained radiologically stable and clinically asymptomatic and did not require a second surgery.

Conclusion: Our study showed that trans-sphenoidal surgery, either with a microscopic or endoscopic approach, was safe for the resection of PitNET. The endoscopic approach has a better outcome in anterior pituitary function improvement. There was no difference in the extent of resection and visual improvement. Postoperative meningitis risk was equally low, and there was no mortality in our transsphenoidal series.

## Introduction

The history of surgery for pituitary neuroendocrine tumour (PitNET, also known as pituitary adenoma) is a fascinating journey that has evolved over centuries. Neurosurgeons initially approached these tumours transcranially through a craniotomy.

Trans-sphenoidal surgery gives surgeons access to the pituitary fossa through the nasal cavity without going through a craniotomy. The roots of trans-sphenoidal surgery can be traced back to the early 20th century. The first documented trans-sphenoidal surgery was performed in 1907 by the Austrian neurosurgeon Hermann Schloffer [1]. Two prominent pituitary surgeons, Harvey Cushing from the United States in 1909 and Oskar Hirsch from Vienna, Austria in 1910, popularised the trans-sphenoidal route [2,3]. Cushing’s apprentice, the Scottish surgeon Norman Dott [4], achieved further evolutions and modifications.

Three decades later, the knowledge was further refined by Gerard Guiot, who began the trans-sphenoidal approach in Paris in 1957 [5]. In the 1960s, a visiting fellow from Montreal, Jules Hardy, met Guiot, became interested in the technique, brought back the knowledge, and continued performing operations using preoperative encephalography and intraoperative radiofluoroscopy. However, the limited visualisation of the tumour caused incomplete tumour removal. In 1965, he resolved the issue by becoming the first surgeon to use an operating microscope and did microscopic trans-sphenoidal surgery [6]. 

Hirschmann performed the first paranasal endoscopy in 1901, using a modified cystoscope to inspect the maxillary sinus [7]. Endoscopy was initially used as an adjunct during microscopic surgery, allowing a wider view of anatomical structures typically hidden from the microscope. This revolution was continued by Apuzzo et al. in 1977 and Bushe and Halves in the late 1970s [8,9].

In the 1990s, a French otolaryngologist, Roger Jankowski from the Central Hospital of the University of Nancy, fully utilised the endoscopic technique for trans-sphenoidal surgery. Jankowski et al. presented the outcome of a three-case series in the 1990s [10]. In the same decade, Jho and Carrau from the University of Pittsburgh Medical Center reported the outcome of 50 patients, 46 of whom were treated via a pure endoscopic approach [11].

The endoscopic approach gained popularity with time due to the panoramic multi-angled view that provides improved visualization. However, the endoscopic technique has limitations in terms of a two-dimensional view, less focus capacity, and a steep learning curve for neurosurgeons [12].

The microscopic approach may be more readily available in many neurosurgical centers than the endoscopic approach, and neurosurgeons may be familiar with and comfortable using a microscope. The cons of the microscopic approach are limited visualisation and a narrow field of view [12].

Various studies have compared the microscopic and endoscopic approaches, and the results are inconclusive regarding which technique is better. Many studies revealed that the endoscopic technique shows promising results on gross tumour resection [12], postoperative pituitary function [13], visual field changes [14], and duration of surgery [15,16]. At the same time, some demonstrated similar outcomes in both endoscopic and microscopic pituitary surgery [17,18].

Two recent meta-analyses of endoscopic versus microscopic trans-sphenoidal surgery for functioning and non-functioning PitNET were reviewed. Guo et al., 2021, found that the endoscopic trans-sphenoidal group was related to a higher incidence of gross-total resection in functioning and non-functioning PitNET. Higher rates of visual improvement and lower rates of meningitis were observed in functioning PitNET following endoscopic trans-sphenoidal resection [19].

Chen et al. (2022) found that endoscopic trans-sphenoidal surgery did not significantly improve gross-total resection and hormone-excess secretion remission. However, it could decrease the rate of diabetes insipidus and septal perforation without increasing the rate of other complications [20]. 

Outcomes for trans-sphenoidal surgical resection of PitNET were heterogeneous. Our work aimed to develop local data on the outcomes of trans-sphenoidal surgery. Local data is crucial as the results of analyses from other countries might overlook some unique local factors, including patient socioeconomic status, logistical problems, resource allocation in public hospitals, and cost-effectiveness.

Despite a paradigm shift towards endoscopic surgery, not all neurosurgical centres in our local setting are equipped with an endoscopic set and a trained skull base ENT surgeon. On the other hand, the microscope is considered an essential asset for all neurosurgical centres. Furthermore, the geographical factor and the travel distance in Sarawak make timely transfer to specialised centres challenging. Microscopic trans-sphenoidal surgery plays a role in delivering pituitary care in neurosurgical centres without an endoscopic facility. This study aimed to evaluate the outcome of microscopic versus endoscopic approach including vision, anterior pituitary function, posterior pituitary function, the volume of residual tumours, and rate of re-surgery.

## Materials and methods

This is a retrospective institutional data review and analysis of PitNET patients who underwent microscopic transnasal or endoscopic endonasal trans-sphenoidal surgery at Sarawak General Hospital from 2014-2021. Patients were identified via the computerized operating theatre documentation system (COTDS). Data were retrieved from the medical record, and MRI films were obtained from the radiology department. This study was approved by the Medical Research and Ethics Committee (MREC), Ministry of Health (MOH) Malaysia [NMRR ID-22-00543-TCY(IIR)].

All adult patients with functioning or non-functioning PitNET who underwent trans-sphenoidal surgery at Sarawak General Hospital from 2014 to 2021 were included. Redo cases were excluded. Sellar tumours with histopathology other than PitNET were excluded.

A total of 60 patients were identified and divided into either microscopic or endoscopic groups. As this was a retrospective study, the choice of surgical approach was not protocol-driven. The decision was made by the treating neurosurgeon based on routine clinical judgement, the resources available at the time of surgery and the patient preference. 

Pre- and postoperatively, patients were subjected to endocrinological and neuro-ophthalmological assessments. Pre- and postoperative otorhinolaryngeal assessments were done for all endoscopic cases. Follow-ups for patients were conducted for at least one year.

Operative procedures

A neurosurgeon solely performed the operative procedure for microscopic trans-sphenoidal surgery, while an ENT surgeon assisted the endoscopic trans-sphenoidal surgery.

One dose of antibiotic with primarily gram-positive coverage was given routinely during induction. The patient’s head was pinned with a 3-pin head clamp for image-guided surgery (IGS). The midfacial area was cleaned with povidone-iodine and draped triangularly with towels. The nostrils were packed with gauze soaked with Moffett’s solution. The surgeon operated on the right side of the patient.

A single nostril approach was utilised for the microscopic transnasal technique. A hemi-transfixion incision was made. Scissors and Cottle's elevators were used to elevate the mucosa to develop a sub-mucoperichondrial plane and dissect posteriorly to reveal the quadrangular septal cartilage, the perpendicular plate of the ethmoid, and vomer. The nasal speculum is used to keep the mucosal tunnel open. The cartilaginous septum is fractured towards the contralateral side. Dissection proceeded posteriorly until the sphenoid prow was identified. Hardy speculum inserted to sphenoid prow. With IGS, the correct sagittal plane is confirmed. Sphenoid septum and mucosa removed. The sella floor was opened using a chisel and mallet, exposing the dura, and the opening was then enlarged with a Kerrison rongeur. A dura incision was made in a cruciate manner. The tumour was removed with standard instruments such as ring curettes, dissectors, suction, and micro forceps. The sella defect was reconstructed with sandwich layers of oxidized cellulose and fat and overlaid with bony fragments. The nasal mucosa was sutured with Vicryl 4/0, and nasal packing was inserted to support the skull base reconstruction.

Both nostrils were utilised for the endoscopic endonasal approach. An ENT surgeon facilitated the surgery. Right middle turbinectomy was done to give more space. The nasoseptal flap was created. After the creation of the nasoseptal flap, sphenoidotomy was done using a microdebrider. During the sellar phase, the sellar floor was removed. IGS was used to confirm the adequacy of the opening. The neurosurgeon continued the surgery while the ENT surgeon was holding the endoscope. The dura mater was opened, and the tumour was removed using gentle suction, a ring curette, and a tissue grasper. 

After tumour removal and haemostasis, the sellar reconstruction was performed by the ENT surgeon using surgical, DuraGen (Integra LifeSciences, Princeton, NJ, USA), Tisseel (Baxter Healthcare Corporation, Deerfield, IL, USA) and nasoseptal flap. Nasal packing was inserted to support the skull base reconstruction. 

In arachnoid breach cases, reconstruction was done using a layer of fat, fascia lata, DuraGen, nasoseptal flap, and Tisseel. Intraoperatively, if there was a CSF leak, a lumbar drain was inserted prior to the reversal of general anaesthesia.

Data collection

All included surgeries were performed or supervised by a senior consultant neurosurgeon. Surgeries were done in a single centre using the same operating room. Each endoscopic endonasal surgery was paired with the same consultant ENT surgeon. The duration of surgery and complications (intraoperative CSF leakage, infection, intracranial haemorrhage, and the need for additional surgery) were recorded. 

Knosp and Hardy's classifications were used to classify adenoma extension based on the patient's pre-operative MRI (1.5T) [21,22]. Volumetric assessments of the tumours pre-and post-surgery were analysed by a radiologist blinded to the method of surgery. Tumour volume (cm3) was calculated using the (A cm x B cm x C cm)/2 formula where A = the largest diameter in axial sections of T1-weighted MRI images with contrast; B = the largest diameter in the sagittal section; and C = the largest diameter in the coronal section [23]. The same method will be used to calculate the volume of tumour remnants on the routine follow-up MRI scans performed three to six months after surgery. 

Following the publications by Cappabianca et al., three degrees of postoperative resection were defined: total resection (when no remaining tumour is observed on MRI scans three months after surgery), subtotal resection (more than 80% of tumour resection compared with pre-operative imaging), and partial resection (less than 80% tumour resected) [24].

In endoscopic endonasal cases, an ENT surgeon evaluated patients pre-and postoperatively. A nasoscope confirmed the healing of the nasoseptal flap. 

Patients were evaluated by an endocrinologist preoperatively. Postoperatively, the patient was re-evaluated by the endocrinology team for pituitary dysfunction. Pituitary hormones were assessed pre-operatively and six weeks post-operatively. Recorded biochemical data included serum growth hormone (GH), insulin-like growth factor 1 (IGF-1), follicle-stimulating hormone (FSH), luteinizing hormone (LH), thyroid function tests (TFT), cortisol, and prolactin. Deficiency refers to biochemical data outside reference values for the specific hormone or if the patient was already on substitution therapy. Intact function refers to hormone levels within the normal range. Increased values of IGF-1, cortisol, or T4 led to specific tests for secreting adenomas. 

Vision was evaluated pre- and postoperatively in all patients who were able to cooperate. Visual acuity was examined using the Snellen chart, and the visual field was documented by the quadrant affected. Visual assessment was performed again at one and three months postoperatively. The visual functions were described as ‘improved,’ ‘static,’ or ‘worse.’

Statistical analysis

Data were cleaned, explored, and analysed using SPSS version 26.0 (IBM Corp., Armonk, NY, USA). Descriptive statistics were used to describe the characteristics of the participants. The distribution of the continuous data was explored using skewness, kurtosis, and histogram. Continuous data were presented as mean ± standard deviation if the data were normally distributed, otherwise median (25th percentile, 75th percentile). Categorical variables were presented as frequency and percentage.

The comparison in various outcomes between microscopic and endoscopic trans-sphenoidal PitNET surgery was performed using the Mann-Whitney U test, Pearson Chi-squared test, and Fisher's exact test. A P value of less than 0.05 indicated statistical significance.

## Results

The demographic characteristics of the participants are presented in Table 1. A total of 60 patients were recruited into the study with a mean age of 54 years (SD: 14.40), with 51.7% (n=31) of them being male patients. Fifteen (25.0%) patients had a functioning adenoma, with three reported cases of prolactin-secreting tumors, 10 of growth hormone, and one case each of adrenocorticotropic hormone and thyroid-stimulating hormone. Most were at Knosp classification of 1 (37.5%, n=21) [21] and Hardy classification of B (51.8%, n=29). Thirty-five patients (58.3%) underwent microscopic approach, while the remaining 25 (41.7%) had an endoscopic approach. The reported presentations include apoplexy (1.7%, n=1), hormone dysfunction (41.7%, n=25), visual disturbance (58.3%, n=35), headache (16.7%, n=10), and incidental finding (8.3%, n=5).

**Table 1:** Summary of characteristic of 60 patients with pituitary neuroendocrine tumour (PitNET) PRL: prolactin, GH: growth hormone, ACTH: adrenocorticotropic hormone, TSH: thyroid-stimulating hormone Knosp grading and Hardy suprasellar extension criteria data were available for 56 patients only because of incomplete radiological data in four patients. Percentages of these variables were calculated based on the available cases. Knosp criteria [21], Hardy suprasellar extension criteria [22]

	Category	Output
Age in years, mean ± SD		54.08±14.40
Gender, n (%)	Male	31 (51.7)
	Female	29 (48.3)
Size, n (%)	Micro	1 (1.7)
	Macro	54 (90.0)
	Giant	5 (8.3)
Adenoma subtype, n (%)	Non-functioning	45 (75.0)
	Functioning	15 (25.0)
If functioning, n (%)	PRL	3 (20.0)
	GH	10 (66.7)
	ACTH	1 (6.7)
	TSH	1 (6.7)
Knosp, n (%)	0	8 (14.3)
	1	21 (37.5)
	2	17 (30.4)
	3	5 (8.9)
	4	5 (8.9)
Hardy suprasellar extension, n (%)	A	10 (17.9)
	B	29 (51.8)
	C	10 (17.9)
	D	7 (12.5)
Approach, n (%)	Microscopic	35 (58.3)
	Endoscopic	25 (41.7)
Presentation, n (%)	Apoplexy	1 (1.7)
	Hormone	25 (41.7)
	Visual	35 (58.3)
	Headache	10 (16.7)
	Incidental	5 (8.3)

The type and grade of tumour in each approach are presented in Table 2. No statistical difference was observed in the ‘level of difficulty’ of the tumour in either approach. Therefore, there is no bias in the analysis of the outcomes of the two approaches.

**Table 2:** Association between approach with type of tumour, Knosp grade and suprasellar extension grade ᵃ Pearson chi-squared test; ᵇ Fisher's exact test PRL: prolactin, GH: growth hormone, ACTH: adrenocorticotropic hormone, TSH: thyroid-stimulating hormone Knosp criteria [21], Hardy suprasellar extension criteria [22]

Characteristic	Overall	Microscopic	Endoscopic	P value
Type of tumour				
Non-functioning	45 (75.0)	28 (80.0)	17 (68.0)	0.290ᵃ
Functioning	15 (25.0)	7 (20.0)	8 (32.0)	
If Functioning				
PRL	3 (20.0)	3 (42.9)	0 (0.0)	0.161ᵇ
GH	10 (66.7)	4 (57.1)	6 (75.0)	
ACTH	1 (6.7)	0 (0.0)	1 (12.5)	
TSH	1 (6.7)	0 (0.0)	1 (12.5)	
KNOSP grade				
0	8 (14.3)	4 (12.9)	4 (16.0)	0.236ᵇ
1	21 (37.5)	12 (38.7)	9 (36.0)	
2	17 (30.4)	7 (22.6)	10 (40.0)	
3	5 (8.9)	5 (16.1)	0 (0.0)	
4	5 (8.9)	3 (9.7)	2 (8.0)	
Hardy suprasellar extension				
A	10 (17.9)	3 (9.7)	7 (28.0)	0.371ᵇ
B	29 (51.8)	18 (58.1)	11 (44.0)	
C	10 (17.9)	6 (19.4)	4 (16.0)	
D	7 (12.5)	4 (12.9)	3 (12.0)	

The overall outcomes of patients who underwent microscopic or endoscopic trans-sphenoidal surgery are presented in Table 3.

**Table 3:** Outcome analysis of 60 patients with pituitary neuroendocrine tumour (PitNET) ᵃ Mann-Whitney U test; ᵇ Pearson chi-squared test; ᶜ Fisher's exact test ICA: internal carotid artery

Outcome	Overall	Microscopic	Endoscopic	P value
Pre-op tumour volume, median (IQR)	3.96 (1.89, 8.31)	3.96 (2.19, 10.09)	2.54 (1.81, 7.23)	0.432ᵃ
Post-op tumour volume, median (IQR)	0.19 (0.00, 1.03)	0.13 (0.00, 2.09)	0.28 (0.00, 0.59)	0.330ᵃ
Complete resection				
Yes	22 (38.6)	11 (34.4)	11 (44.0)	0.459ᵇ
No	35 (61.4)	21 (65.6)	14 (56.0)	
Intra-operative arachnoid breach				
No	46 (76.7)	31 (88.6)	15 (60.0)	0.010ᵇ
Yes	14 (23.3)	4 (11.4)	10 (40.0)	
Diabetes insipidus				
No	52 (86.7)	33 (94.3)	19 (76.0)	0.057ᶜ
Temporary	8 (13.3)	2 (5.7)	6 (24.0)	
Anterior pituitary gland dysfunction				
New	3 (5.0)	1 (2.9)	2 (8.0)	0.006ᶜ
Improved	24 (40.0)	9 (25.7)	15 (60.0)	
Static	33 (55.0)	25 (71.4)	8 (32.0)	
Visual improvement				
Improved	39 (65.0)	24 (68.6)	15 (60.0)	0.493ᵇ
Static	21 (35.0)	11 (31.4)	10 (40.0)	
ICA injury				
No	60 (100.0)	35 (100.0)	25 (100.0)	-
Yes	0 (0.0)	0 (0.0)	0 (0.0)	
CNS infection				
No	58 (96.7)	34 (97.1)	24 (96.0)	>0.950ᶜ
Yes	2 (3.3)	1 (2.9)	1 (4.0)	
Length of surgery in mins, median (IQR)	250 (210, 300)	250 (210, 300)	255 (208, 304)	0.983ᵃ
Length of stay in days, median (IQR)	8.0 (7.0, 10.0)	8.0 (7.0, 10.0)	8.5 (8.0, 10.8)	0.450ᵃ
Nasal symptom post operation				
No	57 (95.0)	34 (97.1)	23 (92.0)	0.565ᶜ
Yes	3 (5.0)	1 (2.9)	2 (8.0)	
Need for second surgery				
No	52 (86.7)	29 (82.9)	23 (92.0)	0.449ᶜ
Yes	8 (13.3)	6 (17.1)	2 (8.0)	
Need for radiotherapy				
No	59 (98.3)	34 (97.1)	25 (100.0)	>0.950ᶜ
Yes	1 (1.7)	1 (2.9)	0 (0.0)	

Complete tumour excision was achieved in 11 (34.4%) patients in the microscopic group and 11 (44.0%) patients in the endoscopic group (Pearson chi-square test, P=0.459, statistically not significant). Further analysis (Table 4) showed that the completeness of resection was affected by the degree of cavernous sinus invasion and suprasellar extension (SSE). A significant association was found between the extent of resection with Knosp grade (P=0.013) and SSE (P= 0.021). A higher proportion of patients with lower Knosp grade and SSE had complete resection.

**Table 4:** Factors associated with the extent of resection Fisher's exact test Knosp criteria [21]

Factors	Extent of resection, n (%)			P value
	Total resection (n = 22)	Subtotal resection (n = 20)	Partial resection (n = 11)	
Knosp grade				
0	5 (71.4)	1 (14.3)	1 (14.3)	0.013
1	13 (61.9)	6 (28.6)	2 (9.5)	
2	4 (26.7)	8 (53.3)	3 (20.0)	
3	0 (0.0)	2 (40.0)	3 (60.0)	
4	0 (0.0)	3 (60.0)	2 (40.0)	
Suprasellar extension				
A	6 (60.0)	2 (20.0)	2 (20.0)	0.021
B	15 (55.6)	8 (29.6)	4 (14.8)	
C	1 (11.1)	6 (66.7)	2 (22.2)	
D	0 (0.0)	4 (57.1)	3 (42.9)	

In the microscopic group, the median operative time was 210 min (IQR 210min, 300 min). In the endoscopic group, the median operative time was 255 min (IQR 208min, 304min) (Mann-Whitney U test, P = 0.983, statistically insignificant). 

Most patients (58.3%) presented with vision impairment in our study. Overall, 65% (n=39) had visual improvement after surgery, and the remaining patients (35%, n=21) had no change in their vision. None of them reported visual deterioration. No statistical difference was discovered in either approach for visual outcome.

We then examined the hormonal improvement. Most patients (86.7%, n=52) had no diabetes insipidus after surgery. Only 13.3% (n=8) experienced diabetes insipidus. 5.7% (n=2) of them came from the microscopic group, while 24.0% (n=6) were from the endoscopic group. Fisher's exact test was P=0.057 (statistically not significant). A higher proportion of patients with improved anterior pituitary gland function was observed in the endoscopic group (25.7% vs 60.0%), with a Fisher's exact test of P=0.006 (statistically significant). 

CSF leakage is a well-known challenge for trans-sphenoidal surgery. The endoscopic group was observed to have a higher intra-operative arachnoid breach, 40% (n=10) vs. 11.4% (n=4) in the microscopic group (Pearson chi-square test, P=0.010, statistically significant). However, CNS infection was low in both endoscopic and microscopic groups. Overall three-point three percent (3.3%, n=2) of patients had meningitis due to cerebrospinal fluid leak (2.9%, n=1 vs 4.0%, n=1) (Fisher's exact test P>0.950, statistically insignificant). Reoperation for CSF leak was performed for one patient in the microscopic group. There was no mortality in our series.

Notably, no case had an arterial injury or venous bleed that caused the surgery to be aborted. Neither approach resulted in extraocular muscle palsy due to oculomotor, trochlear, or abducen nerve injury. 

In the microscopic group, the median length of hospital stay was eight days (IQR 7 days, 10 days). In the endoscopic group, it was 8.5 days (IQR 8 days, 11 days) (Mann-Whitney U test, P=0.450, statistically insignificant). 

In longer follow-up for those patients with residual tumour, only six (17.1 %) patients had second reoperation due to symptomatic tumoural growth. The other 29 (82.9%) patients remained asymptomatic and had no radiological evidence of growth throughout one year follow-up. In addition to the sentinel surgery analysed previously, one (2.9%) patient in the microscopic group and none (0%) in the endoscopic group needed additional radiation (P>0.950) after the second surgery.

## Discussion

Both the microscope and endoscope have been used successfully to approach the sellar region for various pathologies. In our study, we only included PitNET. The rationale was that other pathologies have different consistency and behaviour, which may lead to bias in outcome analysis. 

The microscope is a viable instrument that maintains stereoscopic vision. Most neurosurgeons are used to microscopes and do not face the steep learning curve compared to the endoscopic approach. Conversely, the endoscopic approach provides a broader panoramic visualization and the ability to look around the anatomical corner using an angled endoscope. However, the endoscopic approach requires cooperation with a trained ENT skull base surgeon with endoscopes and a camera system. Not all neurosurgical centers in Malaysia are equipped with endoscopy equipment. 

The analysis conducted in this study found no difference in the extent of resection between the microscopic and endoscopic groups. Various studies reported the same finding. These include studies done by Zaitun et al. (microscopic 30.6% and endoscopic 16.7%, p=0.500) [25], Zaidi et al. (microscopic 81.3% and endoscopic 78.2%, p=0.67) [26], and Ahmad et al. (microscopic 77.7% and endoscopic 75%, P=0.852) [27] which showed similar gross total resection in the microscopic group in comparison to the endoscopic group. 

Few studies have indicated that cavernous sinus invasion is a limiting factor for gross total resection in PitNET [28,29]. Our study has a similar finding whereby gross total resection was only achieved in tumours with Knosp grade 0-2. In addition to horizontal lateral cavernous sinus extension, vertical extension to suprasellar is also another separate indicator to predict the resectability of the tumour. In support of this, Erkan et al. reported that the rate of gross total resection is lower in SSE grade B and above [28]. In our study, the gross total resection rate was highest among SSE grades A and B.

The extent of resection data should be interpreted together with pituitary gland function after surgery. This is because aggressive resection may cause new damage to the gland, leading to new hypopituitarism, which may not be worth it in this context. Overall, 61.4% had residual tumour. However, there is a 40% improvement in anterior pituitary gland function. This implies that decompression of the pituitary gland may restore its function in subtotal resection cases. The endoscopic group has a more significant improvement in anterior pituitary gland function. This result affirms earlier reports that demonstrated higher gland preservation in the endoscopic cohort [26].

We observed that the intraoperative arachnoid breach is higher in the endoscopic group, but the overall meningitis risk is low, 3%. The better visualization of the sella with the endoscope encourages more exposure and dissection, leading to higher rate of intraoperative CSF leakage. However, this drawback can be counterbalanced by the improved reconstructive closure through endoscopy. 

We found no difference between the endoscopic and microscopic groups in the proportion of patients with improved vision in terms of visual acuity and visual field after surgery. These findings are comparable to those of Muskens et al. [30]. These similar results are probably explained by the fact that the visual outcome primarily depends on the timing of decompression of the optic apparatus rather than the approach of the surgery itself. 

Limitation 

This is a retrospective study and is prone to information bias. We only employed univariate analysis but did not evaluate relationships between variables as the sample size was relatively small. Future research should recruit more samples for a longer duration for more representative data.

## Conclusions

Our study suggested that both microscopic and endoscopic approaches are safe and similar in efficacy for PitNET surgery. The differences in the extent of resection, visual outcome, and complications were not statistically significant. There was no mortality in our trans-sphenoidal series. This data would enable counselling for individual patients in our local area.
